# The association between sedentary behavior and falls in older adults: A systematic review and meta-analysis

**DOI:** 10.3389/fpubh.2022.1019551

**Published:** 2022-11-11

**Authors:** YueShuai Jiang, Mei Wang, Shuang Liu, Xiao Ya, GuanTing Duan, ZiPu Wang

**Affiliations:** ^1^School of Sports Management and Communication, Capital Institute of Physical Education and Sports, Beijing, China; ^2^School of Dance and Martial Arts, Capital Institute of Physical Education and Sports, Beijing, China; ^3^Department of Physical Education, Beijing International Studies University, Beijing, China; ^4^Department of Physical Education, Beijing No. 2 Middle School, Beijing Economic-Technological Development Area School, Beijing, China; ^5^College of P.E. and Sports, Beijing Normal University, Beijing, China

**Keywords:** fall, sedentary behavior, older adults, meta-analysis, systematic review

## Abstract

**Background:**

It is generally believed that sedentary behavior (SB) increases the risk of falls among older adults, but the evidence for it remains inconsistent and scarce.

**Purpose:**

Our study aims to provide a systematic review and meta-analysis of available evidence regarding the association of SB with falls in older adults.

**Method:**

A comprehensive search strategy was conducted using several online databases from 1906 to March 2022. Cohort studies both concerning the association between SB and falls and involving participants over 60 years old were regarded as eligible for inclusion. Evidence was pooled by a random-effects meta-analysis. Quality assessment for individual studies was performed with the Newcastle–Ottawa Scale (NOS).

**Results:**

Altogether seven publications were identified, and the age of the 24,750 individuals involved ranging from 60 to 99 years old. Overall quality of the included studies was rated as moderate-to-high quality. We found that SB was significantly associated with increased risk of falls compared with non-SB among older adults [Odds ratio (OR) = 1.17, 95% confidence interval (CI): 1.07–1.28; *I*^2^ = 46.90%, *P*_heterogeneity_ = 0.07, random model]. Subgroup analyses that stratified the studies according to NOS score showed significant differences between groups. Subgroup analysis stratified by SB measurement, sample size, region, publication year, and follow-up duration showed no significant differences between groups.

**Conclusion:**

The findings provide reliable support for the hypothesis that sedentary lifestyles are strong predictors of falls among older adults, offering critical indications to develop strategies for fall prevention.

## Introduction

The incidence of falls increases with age, usually due to age-related issues (e.g., impaired posture control, balance, and gait). Falls and fall-related injuries are common for older adults (1), and are the leading causes of morbidity and mortality in older adults (2). Approximately 30% of adults more than 65 years old fall each year (3), and about half of whom suffer from fall-related injuries (e.g., hospitalizations and hip fractures) (2, 4). Asa result, it not only places a huge burden on their families and the healthcare system but also adds to socio-economic pressure, thereby becoming a public health concern (5).

Since the outbreak of the COVID-19, people's range of motion has been more restricted, especially for the elderly, who are even less active than before, which leads to more frequent sedentary behavior (6, 7). Sedentary behavior (SB) refers to any waking behavior featuring an energy expenditure ≤ 1.5 metabolic equivalents in various postures (sitting, reclining, or lying) (8, 9), which is connected with falls among the old (10). As an independent risk factor for health problems (11), SB is typical in older people. Some studies indicate that SB has a detrimental effect on the quality of life (12) and increases the incidence of falls in old people (13). The reasons for this are varied. For instance, SB was related to the reduction of bone mass (14), sarcopenia (15), and muscle weakness (15), which may increase the fall risk in older adults.

Despite the growing interest in the association between SB and fall risks (12), problems occurring in previous studies remain to be solved. First of all, existing evidence remains ambiguous (14), and some arguments are controversial (15, 16). For example, some studies regarding SB and falls indicated that SB could greatly increase the risk of falls (10, 17), while others showed no significant differences (18, 19). Besides, SB includes various behavior that needs to be measured objectively (such as pedometers and accelerometers), which was neglected in previous studies where subjective test methods were adopted, such as self-reported SB time (20) or questionnaires (21). Furthermore, the previous systematic review was conducted based on qualitative research, lacking objective quantitative analysis. Finally, plentiful new studies focusing on SB and falls have been published with significantly larger datasets, demanding reliable evidence summaries, and requiring updated reliable evidence summaries. As a result, the association between SB and falls among the older people needs further discussion. To look into the association, a meta-analysis, the systematic review that summarizes similar results quantitatively, can be conducted. It enlarges the sample size, improves the statistical effectiveness, as well as obtains results based on a comprehensive analysis, especially when the results of previous studies are conflicting. The aim of this review is, therefore, to determine the overall influence of SB on falls in older adults by conducting a meta-analysis.

## Methods

This meta-analysis was conducted following the Cochrane Collaboration Handbook recommendations (22). We use the PRISMA statement to guide our article selection (23) (Supplementary PRISMA Checklist).

### Search strategies and study selection

With no language and publication date restrictions, a strategic literature search was exhaustively performed to identify relevant observational studies regarding the association between SB and falls in older adults, such as Medline (*via* PubMed), EMBASE, Web of Science, Chinese BioMedical Literature Database, China Science and Technology Journal Database, China National Knowledge Infrastructure and WanFang Database (search strategy conducted from their 1906 to March 2022). After combining medical subject heading terms and keywords, Boolean logic operators were used to widen the scope of literature search. The items and their combinations used are as follows: “sedentary behavior,” “physical inactivity,” “older adults,” “falls,” and “sedentary lifestyle.” All specific search strategies are provided in the supplementary search strategy in production forum (Supplementary Table 4).

Recursive research was manually performed to identify potentially relevant literature by screening similar reviews' references and articles in crucial journals that were presented in the form of abstracts. The selection procedure was separately conducted by two investigators. The Endnote X9 software (Thompson ISI Research Soft, Philadelphia, PA) was used to import and manage all citations, and a third specialist got involved when different opinions between the two investigators emerged. Duplicates were deleted automatically and evaluation of the titles and abstracts was carried out respectively by the two authors. Subsequently, a further full-text evaluation was made to ensure the studies' accuracy and integrity.

### Inclusion criteria

The following criteria were used to select studies: (1) The study design involved only cohort studies; (2) PICOS principles were used for inclusion and exclusion of literature; (3) the study population was older adults (≥60 years old); (4) The exposure factors included any type of SB, such as mobility limitation, physical inactivity, screen time, reclining, mobility limitation, seated position, watching TV, card-playing, and sitting. We chose the SB group with the highest level as a reference category when studies reported multiple categories of SB levels; (5) The measurement strategies of SB were either subjective measurement (e.g., structured questionnaire and telephone interview) or objective measurement (e.g., accelerometer).

### Data extraction and quality assessment

All the basic information extracted from the included studies is as follows: publication year, name of the first author, follow-up time, age, sample size, gender ratio, measurement of falls, study design, definition of sedentary factor and covariates of physical activity. When publications did not report essential data, we contacted the first author to obtain detailed data.

We assessed the quality of cohort studies by the Newcastle-Ottawa Scale (NOS) (Supplementary Table 1), which included three major items: subject selection, comparability between observation groups, and outcome assessment (24). A NOS score <6 was assigned to low quality, while those with a score ≥6 were considered high quality. All the studies were rated independently according to the NOS quality criteria by 2 reviewers, and discrepancies were resolved by a third expert.

### Statistical analyses

For all comparisons, we performed a conventional pairwise meta-analysis using random effects (22). For the results presented as dichotomous data, the effect size was calculated using the odds ratio (OR) with 95% confidence interval (CI) to measure group effects (25). OR was transformed logarithmically when we combined effect size, as it did not conform to the normal distribution. Pooled analyses of the effect of SB on falls were performed based on the random effect model (22). We measured heterogeneity using a *P*-value (<0.1 indicates statistical significance) and *I*^2^ statistic with values of 25, 50, and 75% representing low, moderate, and high heterogeneity, separately (26). The publication bias was first judged through a comparison-adjusted funnel plot, followed by a quantitative egger's test to assess whether *P*-values were < 0.05 (27) (**Figure 2**). Additionally, a sensitivity analysis was conducted to exclude the studies with a higher risk of bias. A subgroup analysis was performed to identify potential sources of heterogeneity or to explore statistically significant differences across studies. The items of subgroup analyses were as follows: SB measurement (accelerometer vs. non-accelerometer), total sample size (sample size ≥1,000 vs. sample size <1,000), region (city vs. rural), year of publication (publication year ≥2013 vs. publication year <2013), NOS quality of included studies (score ≥6 points vs. score <6 points), follow-up duration (>1 vs. ≤ 1 year) (**Table 2**). All the data analyses mentioned above were conducted using STATA software version 14.0 (Stata, Corp, College Station, TX, USA).

## Results

### Literature selection and characteristics of included studies

**Figure 1:**
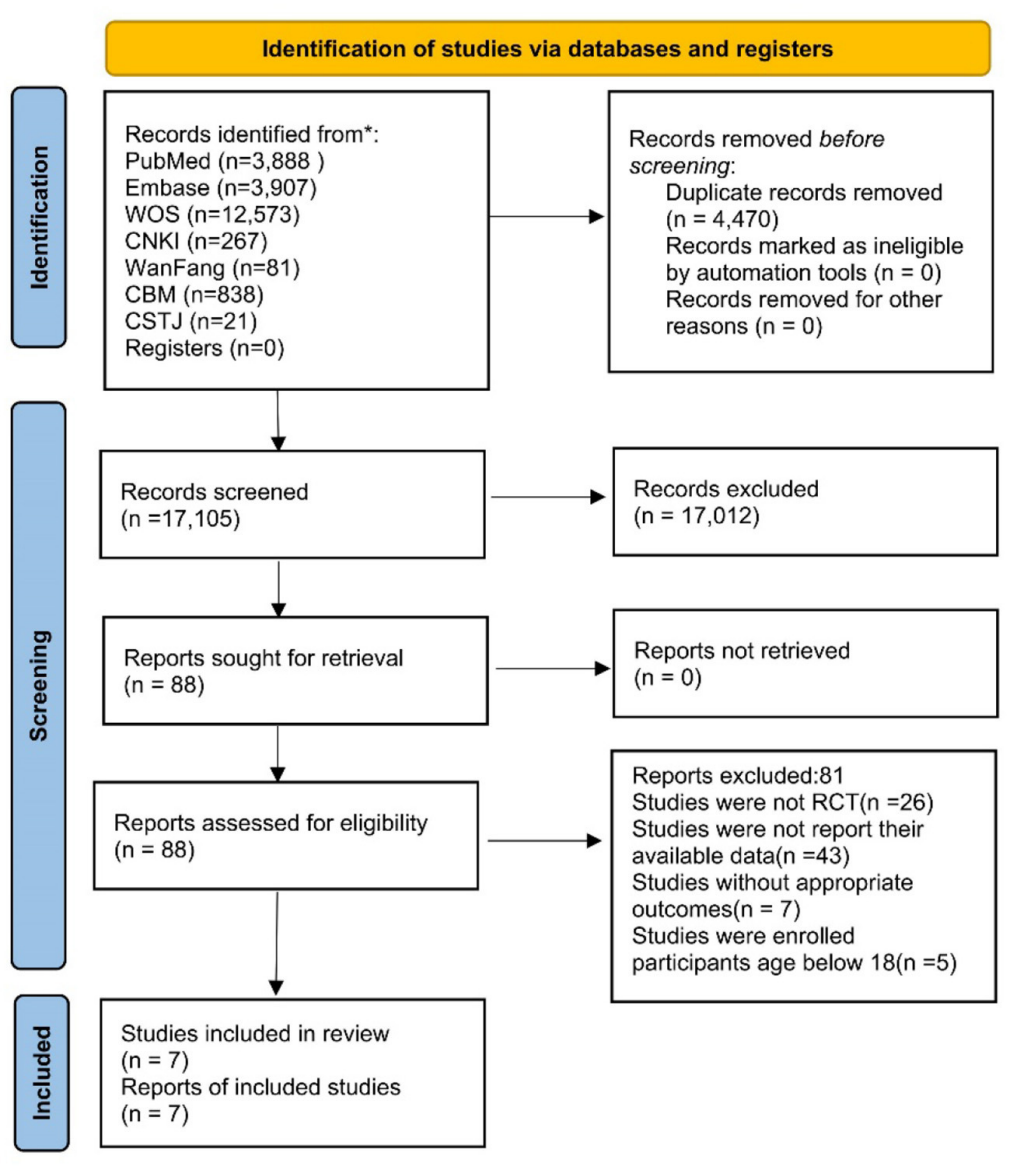
Literature review flowchart. CNKI, China national knowledge infrastructure; CMB, Chinese biomedical; CSTJ, China science and technology journal; WOS, web of science.

The literature search yielded 21,575 studies, among which 3 articles retrieved through reviewing related meta-analysis publications. After excluding 4,470 duplicate references, we further screened the abstracts and titles of the remaining 17,105 articles as they did not meet the inclusion criteria. The remaining 88 articles were scrutinized for full-text screening, and 81 articles were removed due to the availability of data, correlation of outcome indicators, and so on. Finally, seven cohort studies survived the careful selection and qualified for our meta-analysis (10, 19, 28–32). The flow chart depicts the details of the study selection procedure (Figure 1).

All seven included studies enrolled 24,750 individuals aged between 60 and 99 years, among which the number of male participants [5,612 (22.7%)] was significantly lower than that of the female [19,138 (77.3%)]. The follow-up time ranged from 1 to 6 years, with a median follow-up time of 2 years. SB was measured by the accelerometer and various questionnaires such as the international physical activity questionnaire (IPAQ) and women's health initiative physical activity questionnaire (WHIPAQ). Further characteristics of the included studies were summarized in Table 1 (e.g., study design, region, sex proportion, quality of the study, SB assessment, the definition of sedentary factor and covariates of physical activity).

**Table 1:** Demographic characteristics of included studies.

**References**	**Study** **design**	**Number of** **participants**	**Proportion** **of female (%)**	**Age at** **baseline** **(mean ±SD)**	**Region**	**Follow-up** **duration** **(year)**	**NOS** **quality**	**SB assessment**	**Definition of** **sedentary factor**	**Covariates** **of physical** **activity**
Luukinen et al. (31)	CS	1,016	61.00%	76.1 ± 4.9	Finland	2	6	SB was assessed through PQ	Essential daily activity only	NR
Koepsell et al. (29)	CS	1,371	67.60%	NR	USA	2	5	SB was assessed by STI	Physically active (a little)	NR
Cauley et al. (28)	CS	2,731	All male	78.9 ± 5.1	USA	3.5	6	SB was assessed by a biaxial accelerometer	Sedentary activity >1,159.8 min/day	NR
Jefferis et al. (19)	CS	1,655	All male	78 ± 4.5	British	2	7	SB was assessed by ActiGraph GT3x accelerometer over the hip for 1 week	Sedentary time	Duke activity status index
Bea et al. (10)	CS	11,761	All female	50–79	USA	6	8	SB were assessed using WHIPAQ	Physically inactive	NR
Lu et al. (30)	CS	671	41.90%	82.7 ± 3.8	China	1	7	SB was assessed using wrist-worn accelerometer for 7 days	Physical inactivity	NR
Rosenberg et al. (32)	CS	5,545	All female	78.8 ± 6.7	USA	13 months	7	Accelerometers wear at the hip for up to 1 week	Sedentary time >618 min	MVPA

CS, cohort study; MVPA, moderate to vigorous; NR, no report; NOS, newcastle-ottawa scale; PQ, postal questionnaire; STI, screening telephone interview; SD, standard difference; SB, sedentary behavior; WHIPAQ, women's health initiative physical activity questionnaire; y, year.

### Quality of the included studies

Quality assessment of the included studies was done by NOS in Supplementary Table 1. Overall seven studies, six were rated as high quality and the rest one as low quality. The average NOS score was 6.57 points, ranging from 5 to 8 points.

### Primary outcomes

**Figure 2:**
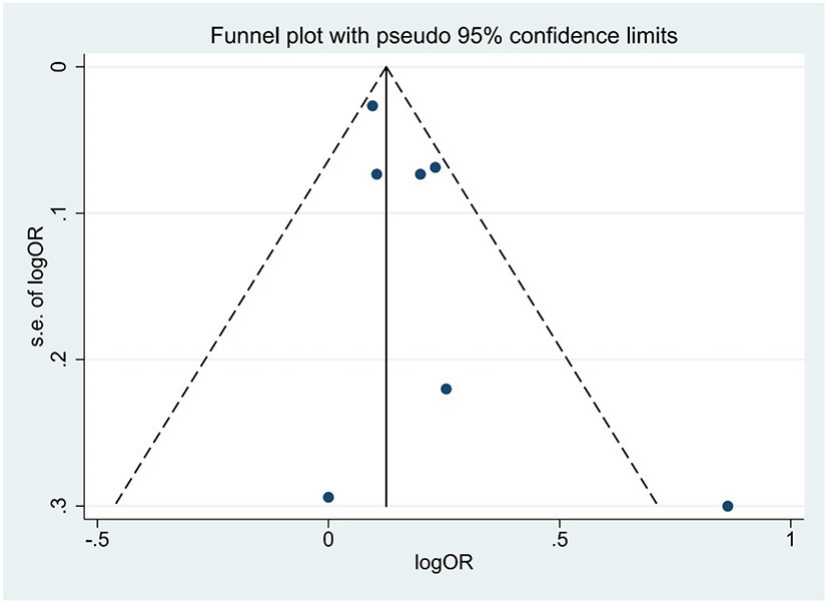
Literature review funnel plot.

Seven studies (24,750 participants) were analyzed to discover the association between SB and falls among older adults. The pooled effect size (OR) was 1.17 (95% CI: 1.07–1.28), with moderate heterogeneity (*I*^2^ = 46.90%, *P* = 0.07, random model) (Table 2), which revealed that older adults with SB were more likely to fall than those without SB. The funnel plot was visually asymmetrical, suggesting the potential existence of publication bias among included studies ($P_{\text{Egge}{\text{r}}^{\text{′}}\text{stest}}$ = 0.11) (Figure 2). PA was a significant variable in the relationship between sedentary and falls in older adults, with two articles adjusted for PA covariates (19, 32) and five articles not adjusted for PA covariates (10, 28–31).

**Table 2:** Primary results and subgroup analyses based on random effect model.

**Subgroup**	**Number of studies**	**Number of participants**	**Odds ratio(95%CI)**	**Heterogeneity**	
				***I*** **^2^ (%)**	***P*** **-value**
Overall	7	24,750	1.17 (1.07–1.28)	46.90	0.07
SB measurement			
Accelerometer	4	10,602	1.28 (1.12–1.48)	35.40	0.20
Non-accelerometer	3	14,148	1.10 (1.04–1.15)	0.00	0.94
Total sample size			
≥1,000	6	24,079	1.13 (1.08–1.18)	4.10	0.39
<1,000	1	671	2.37 (1.31–4.27)	–	–
Region			
City	5	22,363	1.23 (1.09–1.38)	41.50	0.14
Rural	2	2,387	1.09 (1.04–1.15)	0.00	0.74
Publication year			
≥2013	5	22,363	1.23 (1.09–1.38)	41.50	0.14
<2013	2	2,387	1.09 (1.04–1.15)	0.00	0.74
NOS quality			
≥6 points	6	23,379	1.18 (1.08–1.30)	55.10	<0.05
<6 points	1	1,371	1.00 (0.56–1.78)	–	–
Follow-up duration			
>1 year	6	24,079	1.13 (1.08–1.18)	4.10	0.39
≤ 1 year	1	671	2.37 (1.31–4.27)	–	–

CI, confidence interval; NOS, newcastle-ottawa scale; SB, sedentary behavior.

### Subgroup analyses

Based on the primary outcome, the subgroup analyses were conducted with different variables (SB measurement, total sample size, region, publication year, etc.) of interest to explore the possible source of heterogeneity, and most of them yielded consistency that failed to reveal the source of heterogeneity. Nevertheless, when the items of NOS quality were taken into consideration, the NOS score ≥6 (OR = 1.18; 95% CI: 1.08–1.30; *I*^2^ = 55.10%, *P* < 0.05, random model) saw a notable increase of heterogeneity compared with the NOS score <6 (OR = 1.00; 95% CI: 0.56–1.78, random model). Details of pooled effect size for subgroup analyses were based on the random model, respectively, as shown in Table 2.

### Sensitivity analysis

Sensitivity analysis was performed repeatedly by omitting one study each time, with the pooled OR fluctuating between 1.15 (95% CI: 1.05–1.27) and 1.22 (95% CI: 1.09–1.36). It showed that the association between SB and falls changed after the exclusion of three studies (30–32), indicating these three studies may influence our results.

## Discussion

Our study found that older adults with SB present with a higher risk of fall compared to those without, which was consistent with the results of previous studies. We also found that the effect of sedentary behavior on falls in the elderly was not influenced by regional divisions.

This meta-analysis demonstrated that SB can significantly add to the risk of falls in aged people (OR = 1.17; 95% CI: 1.07–1.28). Recent studies also revealed consistent results with ours (10, 32). A longitudinal cohort study, used an accelerometer to measure sedentary time and examine mean sedentary bout duration objectively, which lasted for more than 1 year and involved 5,545 older adults (32). The outcome suggested that longer sedentary time put older women at greater risk of falls. Similarly, in a large sample size cohort study (*n* = 11,761) focusing on SB and falls of postmenopausal women, a logistic regression model was conducted to determine the odds of falling based on the baseline of sedentary time, physical activity duration, and change of physical activity category within 6 years, which showed that SB (0-3METs) was associated with odds of falling (*P* = 0.04), but increasing activity up to ≥9 MET-h/week can also increase the risk of falling (10). Some mechanisms may help explain the relationship between SB and the risk of falls among older adults. First, long-term SB might reduce the physical activity bout duration (10), which cuts down skeletal muscle strength (33) and then lowers the balance performance and gait function (34), resulting in decreased posture control, and consequently leading to a higher risk of falling. In addition, SB often brings about fear of falling (35), a common psychological symptom, and poses a psychological barrier for older adults (36). Some studies have identified the fear of falling as a predictor of falls (35, 37), which can lead to late-life depression (38), reduction of self-efficacy, restriction of social activity (39), and decrease of life-space mobility (40), thereby increasing the fall risks of the aged people. Furthermore, SB might enhance frailty levels of older adults, which contributed to declining cognitive function first (41), and then an increase in fall incidence. Lastly, bone mass might be reduced as a result of SB, and then muscle skeletal pain occurred (42), both of which are triggers of falls.

Based on the subgroup analysis, although older people living in urban areas and those living in rural areas have different lifestyles (43), the results of the impact of sedentary behavior on their falls are consistent. Typically, older people living in cities have better living conditions and they lead a relaxed life under the care of their families (44, 45). Some of the activities they should be doing in this situation are correspondingly reduced, such as household chores or farm work, which leads to less time for physical activity and more time for sedentary activities, which in turn raises the risk of falls (46). And for older adults living in the countryside, they lack awareness of exercise and are unaware of the benefits of regular exercise (47). On the other hand, economic development in rural areas is generally slower and therefore fitness facilities are generally less available, which can also lead to a reduction in physical activity among older people (48). Therefore, despite the different lifestyles of older people in urban and rural areas, the impact of SB on their falls is the same. Although the results showed that the effect of sedentary behavior on falls was not influenced by SB measurements, different sedentary times had varied effects on falls (10), which were greatly influenced by measurement methods (49). Current measures of SB include accelerometers, physical activity scales, and structural interviews, of which accelerometers are the most accurate. Hence, the selection of SB measurement should also be considered in future research. Owing to the moderate heterogeneity (~46.90%), we explored the main factors to explain the heterogeneity. Based on the results of the subgroup analysis, *I*^2^ fluctuated especially in groups of stratification by SB measurement and region. The focus of future studies can be assessment technology to measure SB objectively, such as an accelerometer, a wearable device that enables continuous and precise monitoring of the multiaxial accelerations of body movement in patients. With the help of it, a higher level of reliability can be achieved compared with traditional self-report questionnaires (50). Furthermore, additional studies should be conducted to estimate the dose-response relationship between SB and falls and explore the appropriate time limit for SB.

## Strengths and limitations

There are some strengths in this meta-analysis. For one thing, it is the first meta-analysis ever to discuss the effect of SB on falls in older adults, and the result certifies that SB can significantly increase the incidence of falls. For another, our study employed a comprehensive search strategy and multiple databases, and a complementary search was performed for potential literature such as meetings and abstracts. Consequently, the size of the participants in this study is big enough to provide strong statistical evidence for the estimated effects. Also, this study may act as a useful reference for policymakers, clinicians, or caregivers to make choices and navigate the direction of clinical decision-making, thereby advancing future research and clinical application.

Several limitations should also be acknowledged. First of all, all results came from a relatively limited number of included studies, leading to insufficient evidence in our analysis. Second, the methodological shortcomings of the observational studies might jeopardize the overall quality of the research. Furthermore, nearly a quarter of the participants were male, which may cause some bias. And the heterogeneity and publication bias risk of the included studies was mostly reflected in outcome assessment blinding and selective bias items. In addition, some of the articles' models were not adjusted for PA covariates, potentially reducing the precision and stability of the study results and thus biasing the results of our articles. Last, sedentary behavior (e.g., watching TV) does not always mean low physical activity (e.g., doing housework). Typically, for older adults, a cut point of ≤ 1.5 METs is used to distinguish sedentary behavior from light physical activity (8). Low physical activity and sedentary behavior need to be clearly defined in future studies.

## Conclusions and implications

It is found that there is a positive association between SB and falls among older adults, which serves as an important step forward for considering SB as a modifiable risk factor for falls in older adults. Reducing SB can help reduce the level of falls and improve the quality of life of older adults. Therefore, older adults should be encouraged to reduce sedentary behavior and engage in appropriate physical activity. Considering the quantity and quality of the included studies, our conclusion needs to be interpreted with caution. In addition, given the rising prevalence of falls and universal SB in modern society, the results of our study provide valuable insights into promoting clinical and public health. In the future, more longitudinal studies should be conducted to better demonstrate the relationship between sedentary behavior and falls in older adults.

## Data availability statement

The original contributions presented in the study are included in the article/Supplementary material, further inquiries can be directed to the corresponding author.

## Author contributions

YJ served as the principal author and had full access to all the data in the study, taking responsibility for the analysis and interpretation of data, and the acquisition of the data analysis. XY contributed to the study's conception and design. SL and GD contributed to data acquisition of data. MW contributed to the draft of the manuscript. ZW contributed to the critical revision of the manuscript for important intellectual content. All authors contributed to the article and approved the submitted version.

## Conflict of interest

The authors declare that the research was conducted in the absence of any commercial or financial relationships that could be construed as a potential conflict of interest.

## Publisher's note

All claims expressed in this article are solely those of the authors and do not necessarily represent those of their affiliated organizations, or those of the publisher, the editors and the reviewers. Any product that may be evaluated in this article, or claim that may be made by its manufacturer, is not guaranteed or endorsed by the publisher.
