# Supplementary material for: The association between sedentary behavior and falls in older adults: A systematic review and meta-analysis
Source: Front Public Health. 2022 Nov 11;10:1019551. doi: 10.3389/fpubh.2022.1019551 (PMC9691853; doi:10.3389/fpubh.2022.1019551)
Supplement: Supplementary file 1 [file Table_1.DOCX]

|  | | | | | | | | | | |
| --- | --- | --- | --- | --- | --- | --- | --- | --- | --- | --- |
| **Supplementary Table 1** Methodological quality of the selected studies according to the NOS | | | | | | | | | | |
| Study | Selection | | | |  | Comparability |  | Exposure | | |
|  | Representativeness of the Exposed Cohort | Selection of the Non-exposed Cohort | Ascertainment of Exposure | Outcome of Interest not Presented at Start of Study |  | Comparability of Cohorts on the Basis of the Design or Analysis |  | Assessment of Outcome | Was Follow-up Long Enough for Outcomes to Occur | Adequacy of Follow up of Cohorts |
| Luukinen et al, 1996 | ★ | ★ | ★ | ★ |  | ★ |  | N/A | ★ | N/A |
| Koepsell et al, 2004 | ★ | ★ | ★ | ★ |  | ★ |  | N/A | N/A | N/A |
| Cauley et al, 2013 | N/A | ★ | ★ | ★ |  | ★ |  | ★ | N/A | ★ |
| Jefferis et al, 2015 | N/A | ★ | ★ | ★ |  | ★★ |  | ★ | N/A | ★ |
| Bea, 2017 | ★ | ★ | ★ | ★ |  | ★★ |  | N/A | ★ | ★ |
| Lu et al, 2020 | ★ | ★ | ★ | ★ |  | ★★ |  | N/A | N/A | ★ |
| Rosenberg et al, 2021 | N/A | ★ | ★ | ★ |  | ★★ |  | N/A | ★ | ★ |
| This table identifies “high” quality choices with a “star”. A study can be awarded a maximum of 1 star for each numbered item within the Selection and Exposure categories. A maximum of 2 stars can be given for Comparability. | | | | | | | | | | |
| ★, yes; N/A, Not applicable; NOS, Newcastle-ottawa scale | | | | | | | | | | |
