# Supplementary material for: The association between sedentary behavior and falls in older adults: A systematic review and meta-analysis
Source: Front Public Health. 2022 Nov 11;10:1019551. doi: 10.3389/fpubh.2022.1019551 (PMC9691853; doi:10.3389/fpubh.2022.1019551)
Supplement: Supplementary file 2 [file Table_2.docx]

**Web of science Search Strategy**

#1TS (Sedentary Behavior or sedentary lifestyle or Behavior, Sedentary or Sedentary Behavior* or Sedentary behaviour or Lifestyle, Sedentary or Physical Inactivity or Inactivity, Physical or Lack of Physical Activity or Sedentary Time or Sedentary Times or Time, Sedentary or mobility limitation or reclining or lying or stationary behavior or Screen Time or Screen Time* or Sitting Position or Position, Sitting or Sitting Position* or Sitting or Seated Position or Position, Seated or Position*, Seated or Seated Position* or non-screen-based sedentary time or watching TV or reading or listening music or transportation or play card or using telephone)

#2TS (Aged or elderly or older people older adult* or aged patient or aged people or aged person or aged subject or elderly patient or elderly people or elderly person or elderly subject or senior citizen or senium)

#3TS (Accidental Fall* or fall or Fall* or Falling or Fall*, Accidental or Accidental Fall or Fall, Accidental or Slip and Fall or Fall and Slip or falling)

#1 AND #2 AND #3

**PubMed** **Search Strategy**

#1 ((((((((Accidental Falls[Title/Abstract]) OR (fall[Title/Abstract])) OR (Falls[Title/Abstract])) OR (Falling[Title/Abstract])) OR (Falls, Accidental[Title/Abstract])) OR (Accidental Fall[Title/Abstract])) OR (Fall, Accidental[Title/Abstract])) OR (Slip[Title/Abstract] AND Fall[Title/Abstract])) OR (Fall[Title/Abstract] AND Slip[Title/Abstract])

#2 ((((((((((((((((((((((((((((((((Sedentary Behavior[Title/Abstract]) OR (Behavior, Sedentary[Title/Abstract])) OR (Sedentary Behaviors[Title/Abstract])) OR (sedentary behaviour[Title/Abstract])) OR (Sedentary Lifestyle[Title/Abstract])) OR (Lifestyle, Sedentary[Title/Abstract])) OR (Physical Inactivity[Title/Abstract])) OR (Inactivity, Physical[Title/Abstract])) OR (Lack of Physical Activity[Title/Abstract])) OR (Sedentary Time[Title/Abstract])) OR (Sedentary Times[Title/Abstract])) OR (Time, Sedentary[Title/Abstract])) OR (mobility limitation[Title/Abstract])) OR (reclining[Title/Abstract])) OR (lying[Title/Abstract])) OR (stationary behavior[Title/Abstract])) OR (Screen Time[Title/Abstract])) OR (Screen Times[Title/Abstract])) OR (Sitting Position[Title/Abstract])) OR (Position, Sitting[Title/Abstract])) OR (Sitting Positions[Title/Abstract])) OR (Sitting[Title/Abstract])) OR (Seated Position[Title/Abstract])) OR (Position, Seated[Title/Abstract])) OR (Positions, Seated[Title/Abstract])) OR (Seated Positions[Title/Abstract])) OR (non-screen-based sedentary time[Title/Abstract])) OR (watching TV[Title/Abstract])) OR (reading[Title/Abstract])) OR (listening music[Title/Abstract])) OR (transportation[Title/Abstract])) OR (play card[Title/Abstract])) OR (using telephone[Title/Abstract])

#3 (((((((((((((Aged[Title/Abstract]) OR (elderly[Title/Abstract])) OR (older people[Title/Abstract])) OR (older adults[Title/Abstract])) OR (aged patient[Title/Abstract])) OR (aged people[Title/Abstract])) OR (aged person[Title/Abstract])) OR (aged subject[Title/Abstract])) OR (elderly patient[Title/Abstract])) OR (elderly people[Title/Abstract])) OR (elderly person[Title/Abstract])) OR (elderly subject[Title/Abstract])) OR (senior citizen[Title/Abstract])) OR (senium[Title/Abstract])

#4 (((((((((((((((Aged[Title/Abstract]) OR (elderly[Title/Abstract])) OR (older people[Title/Abstract])) OR (older adults[Title/Abstract])) OR (aged patient[Title/Abstract])) OR (aged people[Title/Abstract])) OR (aged person[Title/Abstract])) OR (aged subject[Title/Abstract])) OR (elderly patient[Title/Abstract])) OR (elderly people[Title/Abstract])) OR (elderly person[Title/Abstract])) OR (elderly subject[Title/Abstract])) OR (senior citizen[Title/Abstract])) OR (senium[Title/Abstract])) AND (((((((((((((((((((((((((((((((((Sedentary Behavior[Title/Abstract]) OR (Behavior, Sedentary[Title/Abstract])) OR (Sedentary Behaviors[Title/Abstract])) OR (sedentary behaviour[Title/Abstract])) OR (Sedentary Lifestyle[Title/Abstract])) OR (Lifestyle, Sedentary[Title/Abstract])) OR (Physical Inactivity[Title/Abstract])) OR (Inactivity, Physical[Title/Abstract])) OR (Lack of Physical Activity[Title/Abstract])) OR (Sedentary Time[Title/Abstract])) OR (Sedentary Times[Title/Abstract])) OR (Time, Sedentary[Title/Abstract])) OR (mobility limitation[Title/Abstract])) OR (reclining[Title/Abstract])) OR (lying[Title/Abstract])) OR (stationary behavior[Title/Abstract])) OR (Screen Time[Title/Abstract])) OR (Screen Times[Title/Abstract])) OR (Sitting Position[Title/Abstract])) OR (Position, Sitting[Title/Abstract])) OR (Sitting Positions[Title/Abstract])) OR (Sitting[Title/Abstract])) OR (Seated Position[Title/Abstract])) OR (Position, Seated[Title/Abstract])) OR (Positions, Seated[Title/Abstract])) OR (Seated Positions[Title/Abstract])) OR (non-screen-based sedentary time[Title/Abstract])) OR (watching TV[Title/Abstract])) OR (reading[Title/Abstract])) OR (listening music[Title/Abstract])) OR (transportation[Title/Abstract])) OR (play card[Title/Abstract])) OR (using telephone[Title/Abstract]))) AND (((((((((Accidental Falls[Title/Abstract]) OR (fall[Title/Abstract])) OR (Falls[Title/Abstract])) OR (Falling[Title/Abstract])) OR (Falls, Accidental[Title/Abstract])) OR (Accidental Fall[Title/Abstract])) OR (Fall, Accidental[Title/Abstract])) OR (Slip[Title/Abstract] AND Fall[Title/Abstract])) OR (Fall[Title/Abstract] AND Slip[Title/Abstract]))

#5 (((((((((Accidental Falls[Title/Abstract]) OR (fall[Title/Abstract])) OR (Falls[Title/Abstract])) OR (Falling[Title/Abstract])) OR (Falls, Accidental[Title/Abstract])) OR (Accidental Fall[Title/Abstract])) OR (Fall, Accidental[Title/Abstract])) OR (Slip[Title/Abstract] AND Fall[Title/Abstract])) OR (Fall[Title/Abstract] AND Slip[Title/Abstract])) AND (((((((((((((((((((((((((((((((((Sedentary Behavior[Title/Abstract]) OR (Behavior, Sedentary[Title/Abstract])) OR (Sedentary Behaviors[Title/Abstract])) OR (sedentary behaviour[Title/Abstract])) OR (Sedentary Lifestyle[Title/Abstract])) OR (Lifestyle, Sedentary[Title/Abstract])) OR (Physical Inactivity[Title/Abstract])) OR (Inactivity, Physical[Title/Abstract])) OR (Lack of Physical Activity[Title/Abstract])) OR (Sedentary Time[Title/Abstract])) OR (Sedentary Times[Title/Abstract])) OR (Time, Sedentary[Title/Abstract])) OR (mobility limitation[Title/Abstract])) OR (reclining[Title/Abstract])) OR (lying[Title/Abstract])) OR (stationary behavior[Title/Abstract])) OR (Screen Time[Title/Abstract])) OR (Screen Times[Title/Abstract])) OR (Sitting Position[Title/Abstract])) OR (Position, Sitting[Title/Abstract])) OR (Sitting Positions[Title/Abstract])) OR (Sitting[Title/Abstract])) OR (Seated Position[Title/Abstract])) OR (Position, Seated[Title/Abstract])) OR (Positions, Seated[Title/Abstract])) OR (Seated Positions[Title/Abstract])) OR (non-screen-based sedentary time[Title/Abstract])) OR (watching TV[Title/Abstract])) OR (reading[Title/Abstract])) OR (listening music[Title/Abstract])) OR (transportation[Title/Abstract])) OR (play card[Title/Abstract])) OR (using telephone[Title/Abstract]))

**Embase Search Strategy**

#11. #3 AND #6

#10. #3 AND #6 AND #9

#9. #7 OR #8

#8. 'elderly':ab,ti OR 'older people':ab,ti OR 'older adults':ab,ti OR 'aged patient':ab,ti OR 'aged people':ab,ti OR 'aged person':ab,ti OR 'aged subject':ab,ti OR 'elderly patient':ab,ti OR 'elderly people':ab,ti OR 'elderly person':ab,ti OR 'elderly subject':ab,ti OR 'senior citizen':ab,ti OR 'senium':ab,ti

#7. 'aged'/exp

#6. #4 OR #5

#5. 'sedentary behavior':ab,ti OR 'behavior, sedentary':ab,ti OR 'sedentary behaviors':ab,ti OR 'sedentary behaviour':ab,ti OR 'lifestyle, sedentary':ab,ti OR 'physical inactivity':ab,ti OR 'inactivity, physical':ab,ti OR 'lack of physical activity':ab,ti OR 'sedentary time':ab,ti OR 'sedentary times':ab,ti OR 'time, sedentary':ab,ti OR 'mobility limitation':ab,ti OR 'reclining':ab,ti OR 'lying':ab,ti OR 'stationary behavior':ab,ti OR 'screen time':ab,ti OR 'screen times':ab,ti OR 'sitting position':ab,ti OR 'position, sitting':ab,ti OR 'sitting positions':ab,ti OR 'sitting':ab,ti OR 'seated position':ab,ti OR 'position, seated':ab,ti OR 'positions, seated':ab,ti OR 'seated positions':ab,ti OR 'non-screen-based sedentary time':ab,ti OR 'watching tv':ab,ti OR 'reading':ab,ti OR 'listening music':ab,ti OR 'transportation':ab,ti OR 'play card':ab,ti OR 'using telephone':ab,ti

#4. 'sedentary lifestyle'/exp

#3. #1 OR #2

#2. 'accidental falls':ab,ti OR 'fall':ab,ti OR 'falls':ab,ti OR (fall:ab,ti AND slip:ab,ti) OR 'falls, accidental':ab,ti OR 'accidental fall':ab,ti OR 'fall, accidental':ab,ti OR (slip:ab,ti AND fall:ab,ti)

#1. 'falling'/exp

The following content is the Chinese database search strategy, the search strategy of Chinese literature can be directly pasted and copied to the URL of Chinese journals for direct search.

**Chinese BioMedical Literature Search Strategy**

1) 意外跌倒 or 跌倒 or 滑倒 or 跌落 13011 2022-03-09 18:42:39.0

2) 久坐 or 久坐行为 or 久坐生活方式 or 缺乏运动 or 久坐时间 or 活动受限 or 斜靠 or 躺 or 视屏时间 or 坐姿 or 看电视 or 阅读 or 听音乐 or 交通 or 打牌

3) (老年 or 年长的 or 老年人 or 老年患者 or 老人 or 年长者) 677859 2022-03-09 18:43:50.0

4) (#3) AND (#2) AND (#1)

**China National Knowledge Infrastructure Search Strategy**

#1=(主题=老年＋年长的＋老年人＋老年患者＋老人＋年长者)

#2=((主题%=老年+年长的+老年人+老年患者+老人+年长者or题名%=老年+年长的+老年人+老年患者+老人+年长者)AND(主题%=久坐+久坐行为+久坐生活方式+缺乏运动+久坐时间+活动受限+斜靠+躺+视屏时间+坐姿+看电视+阅读+听音乐+交通+打牌+手机or题名%m久坐+久坐行为+久坐生活方式+缺乏运动+久坐时间+活动受限+斜靠+躺+视屏时间+坐姿+看电视+阅读+听音乐+交通+打牌+手机))

#3=(((主题%=老年+年长的+老年人+老年患者+老人+年长者or题名%=老年+年长的+老年人+老年患者+老人+年长者)AND(主题%=久坐+久坐行为+久坐生活方式+缺乏运动+久坐时间+活动受限+斜靠+躺+视屏时间+坐姿+看电视+阅读+听音乐+交通+打牌+手机or题名%=久坐+久坐行为+久坐生活方式+缺乏运动+久坐时间+活动受限+斜靠+躺+视屏时间+坐姿+看电视+阅读+听音乐+交通+打牌+手机))AND(主题%=意外跌倒+趺倒+滑倒+跌落or 题名%=意外跌倒+跌倒+滑倒+跌落))

**WanFang** **Search Strategy**

(老年 or 年长的 or 老年人 or 老年患者 or 老人 or 年长者) and 题名或关键词:(久坐 or 久坐行为 or 久坐生活方式 or 缺乏运动 or 久坐时间 or 活动受限 or 斜靠 or 躺 or 视屏时间 or 坐姿 or 看电视 or 阅读 or 听音乐 or 交通 or 打牌) and 题名或关键词:(意外跌倒 or 跌倒 or 滑倒 or 跌落)

**China Science and Technology Journal Search Strategy**

#1=意外跌倒 or 跌倒 or 滑倒 or 跌落

#2=久坐 or 久坐行为 or 久坐生活方式 or 缺乏运动 or 久坐时间 or 活动受限 or 斜靠 or 躺 or 视屏时间 or 坐姿 or 看电视 or 阅读 or 听音乐 or 交通 or 打牌

#3=(老年 or 年长的 or 老年人 or 老年患者 or 老人 or 年长者)
